# Using Machine Learning to Predict MACEs Risk in Patients with Premature Myocardial Infarction

**DOI:** 10.31083/RCM31298

**Published:** 2025-05-20

**Authors:** Jing-xian Wang, Miao-miao Liang, Peng-ju Lu, Zhuang Cui, Yan Liang, Yu-hang Wang, An-ran Jing, Jing Wang, Meng-long Zhang, Yin Liu, Chang-ping Li, Jing Gao

**Affiliations:** ^1^Clinical School of Thoracic, Tianjin Medical University, 300070 Tianjin, China; ^2^Department of Cardiology, Tianjin Chest Hospital, 300222 Tianjin, China; ^3^School of Public Health, Tianjin Medical University, 300070 Tianjin, China; ^4^Cardiovascular Institute, Tianjin Chest Hospital, 300222 Tianjin, China; ^5^Tianjin Key Laboratory of Cardiovascular Emergency and Critical Care, 300222 Tianjin, China; ^6^Chest Hospital, Tianjin University, 300072 Tianjin, China

**Keywords:** acute myocardial infarction, premature myocardial infarction, machine learning, major adverse cardiovascular events, prediction model

## Abstract

**Background:**

The study aimed to develop an interpretable machine learning (ML) model to assess and stratify the risk of long-term major adverse cardiovascular events (MACEs) in patients with premature myocardial infarction (PMI) and to analyze the key variables affecting prognosis.

**Methods:**

This prospective study consecutively included patients (male ≤50 years, female ≤55 years) diagnosed with acute myocardial infarction (AMI) at Tianjin Chest Hospital between January 2017 and December 2022. The study endpoint was the occurrence of MACEs during the follow-up period, which was defined as cardiac death, nonfatal stroke, readmission for heart failure, nonfatal recurrent myocardial infarction, and unplanned coronary revascularization. Four machine learning models were built: COX proportional hazards model (COX) regression, random survival forest (RSF), extreme gradient boosting (XGBoost), and DeepSurv. Models were evaluated using concordance index (C-index), Brier score, and decision curve analysis to select the best model for prediction and risk stratification.

**Results:**

A total of 1202 patients with PMI were included, with a median follow-up of 26 months, and MACEs occurred in 200 (16.6%) patients. The RSF model demonstrated the best predictive performance (C-index, 0.815; Brier, 0.125) and could effectively discriminate between high- and low-risk patients. The Kaplan-Meier curve demonstrated that patients categorized as low risk showed a better prognosis (*p* < 0.0001).

**Conclusions:**

The prognostic model constructed based on RSF can accurately assess and stratify the risk of long-term MACEs in PMI patients. This can help clinicians make more targeted decisions and treatments, thus delaying and reducing the occurrence of poor prognoses.

## 1. Introduction

In recent years, the prevalence and mortality of acute myocardial infarction (AMI) have tended to be younger and are the leading cause of premature death worldwide [1], with about 4%–10% of AMI patients reported to be aged ≤40 or 45 years [2, 3]. The increase of metabolic risk factors in young people, such as obesity, diabetes, high uric acid, and hypertension, has increased the incidence of premature myocardial infarction (PMI) and major adverse cardiovascular events (MACEs) [4], which seriously affect the workability and quality of life of patients, causing a certain burden on families and social economy. Obtaining accurate risk prediction of long-term MACEs after PMI, and therefore early intervention to improve patient prognosis as much as possible, is of utmost importance in clinical management [5, 6, 7, 8].

Machine learning (ML) algorithms provide powerful tools for researchers to learn rules in data and make data-driven outcome predictions by capturing high-dimensional, linear, or non-linear relationships between clinical variables [9]. ML has been used in many medical-related fields, such as diagnosis, outcome prediction, treatment, and medical image interpretation, and is superior to proven traditional risk stratification tools [10, 11, 12, 13, 14]. For example, a study using the American College of Cardiology Chest Pain-MI registry that used an ML model to predict death after AMI reported an area under the curve (AUC) value of close to 0.9 for each ML model, with extreme gradient boosting (XGBoost) provide better risk solutions for high-risk individuals [15]. Another ML-based study of adverse event prediction in acute coronary syndrome (apolipoprotein A1/B, ApoA1/B) showed that different machine learning models showed good predictive performance in predicting all-cause death, myocardial infarction, and major bleeding in acute coronary syndrome (ACS) patients at 1 year after discharge, and compared with traditional risk prediction tools, ML algorithm has advantages in predicting MACEs [16].

Compared with elderly MI patients, young myocardial infarction (MI) patients may have a different risk factor spectrum, and PMI patients often have other unique metabolic risk factors [17]. There are still few studies on the related risk factors affecting the occurrence of AMI adverse events in young adults, and the previous studies using machine learning algorithms to establish MACEs prediction models in young MI patients are also limited. Therefore, the development of machine learning predictive models for these patients to guide early clinical intervention has certain research value. In summary, the purpose of this study was to use machine learning algorithms to assess and stratify the risk of long-term MACEs in PMI patients, and to analyze key clinical variables affecting the occurrence of MACEs.

## 2. Materials and Methods

### 2.1 Study Cohort

**Fig. 1.**
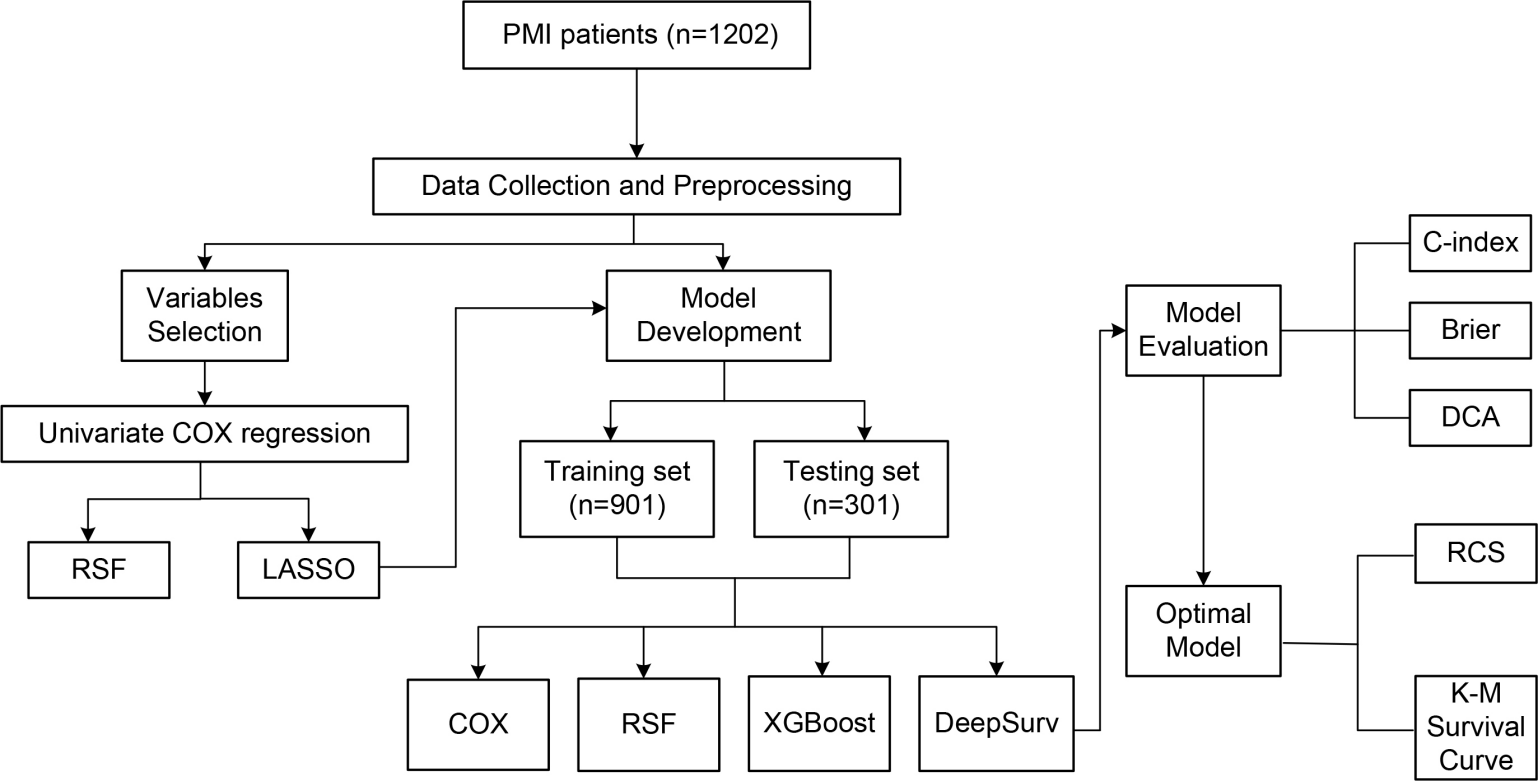
**Flowchart**. PMI, premature myocardial infarction; RSF, random survival forest; LASSO, least absolute shrinkage and selection operator; DCA, decision curve analysis; XGBoost, extreme gradient boosting; RCS, restricted cubic spline; COX, COX proportional hazards model; K-M, Kaplan-Meier; C-index, concordance index.

The flow of the study is shown in Fig. 1. This is a single-center, prospective, observational cohort study. Consecutive patients admitted to Tianjin Chest Hospital for AMI between January 2017 and December 2022, meeting the PMI age threshold, were included in the PMI cohort.

Inclusion criteria:

(1) Age >18 years old, female age ≤55 years old, male age ≤50 years old;

(2) Meet the diagnostic criteria of AMI. The diagnosis of AMI in this study was based on the fourth Global Definition of Myocardial Infarction [18]. That is, elevation of serum myocardial markers (primarily troponin) above at least 99% of the reference limit, accompanied by at least one of the following clinical symptoms:

① Typical symptoms of myocardial ischemia (persistent chest pain >30 minutes, not relieved by taking 1–2 nitroglycerin tablets, accompanied by sweating, nausea, vomiting, pallor, and other symptoms);

② New ischemic electrocardiogram (ECG) changes (including increased T wave width, new ST segment and T wave (ST-T) changes, or left bundle branch block), ECG pathological Q-wave formation;

③ Imaging evidence showed new local wall motion abnormalities;

④ Coronary angiography confirmed thrombus in the coronary artery.

Coronary angiography (CAG) was performed by two or more cardiologists qualified in coronary diagnosis and treatment at our center.

Exclusion criteria:

(1) Patients with severe liver and/or renal failure;

(2) Patients with congenital heart disease and/or valvular heart disease;

(3) Patients with severe inflammatory diseases and/or malignant tumors;

(4) Patients with missing transthoracic echocardiography and/or other data;

(5) Patients without signed informed consent.

The study followed the Declaration of Helsinki, was approved by the Ethics Committee of Tianjin Chest Hospital (No. 2017KY-007-01), and written informed consent was obtained from all participants.

### 2.2 Data Collection

Establish the electronic medical record database of PMI patients in our center. The Epidata data entry system uses a two-person entry method, and to ensure data quality, all event diagnoses are further verified by the review of the medical records by two cardiologists. The lead researcher, statistician, and other team members collaborate to review the data to ensure accuracy, completeness, and reliability.

Data collected included general characteristics, including gender, age, body mass index (BMI), personal history (smoking and drinking history), previous medical history [diabetes, hypertension, hyperlipidemia, chronic kidney disease (CKD), and stroke history], family history of coronary artery disease (CAD), and type of AMI; admission vital signs (heart rate, blood pressure, shock index); laboratory tests [blood routine, liver and kidney function, coagulation function, fasting blood glucose, lipids, brain natriuretic peptide (BNP), peak creatine kinase MB (CK-MB), peak value cardiac troponin T (TNT)], CAG including diseased vessels, number of diseased vessels, coronary thrombosis, percutaneous coronary intervention (PCI), complete occlusion, Syntax score, and transthoracic echocardiography (TTE) parameters (left atrial diameter, left ventricular diameter, left ventricular ejection fraction). Peak CK-MB and TNT levels were recorded, and remaining laboratory parameters were measured after a rapid overnight stay (≥8 hours) on the day of admission. The Syntax score [19] was used to assess the severity of CAD and to assist CAD patients with risk stratification and revascularization strategies. It is calculated using online software version 2.28 (https://syntaxscore.org/). Using Killip ≥II as the cutoff value, Killip ≥II indicates clear evidence of heart failure (e.g., pulmonary rales or elevated jugular venous pressure). Compared to Killip I patients, these patients have significantly worse prognoses and receive greater attention in clinical management and interventions. In addition, we documented patients’ medication during hospitalization, including antiplatelet drugs, statins, diuretics, angiotensin-converting enzyme inhibitors (ACEIs), angiotensin receptor blockers (ARBs), and beta blockers.

### 2.3 Study Endpoint

The endpoint of the study was the occurrence of MACEs during follow-up, including cardiac death, nonfatal stroke, readmission for heart failure, nonfatal recurrent myocardial infarction, and unplanned coronary revascularization. All patients were followed up after discharge by a trained specialist on an outpatient basis or by telephone to record the occurrence of MACEs in PMI patients during the follow-up period. Cardiac death was mainly caused by sudden cardiac death, acute congestive heart failure, acute myocardial infarction, severe arrhythmia, and other structural/functional heart disease. Stroke is defined based on imaging findings or typical symptoms. According to the guidelines of the European Society of Cardiology, the diagnosis of heart failure refers to the ventricular filling and/or ejection function impairment caused by various cardiac structural or functional diseases, and the cardiac output cannot meet the metabolic needs of the body tissues, resulting in clinical manifestations such as dyspnea, limited physical activity, and fluid retention. AMI was diagnosed comprehensively based on the results of chest pain, myocardial enzyme pattern changes, and electrocardiogram [18]. Unplanned coronary revascularization is defined as revascularization driven by ischemic symptoms or any pathological event, including unplanned PCI and coronary artery bypass grafting (CABG).

### 2.4 Model Construction and Evaluation

#### 2.4.1 Data Preprocessing

The study initially included 75 clinical variables. The variables with a deletion rate of more than 10% were deleted, the variables with a deletion rate of less than 10% were filled with multiple imputation methods, and 70 clinical variables were finally included. Multiple imputation was performed using the R 4.4.1 software (R Core Team, Auckland, New Zealand) (mice package). The number of imputations was set to 5 (m = 5), with a maximum of 10 iterations (maxit = 10). The predictive mean matching (PMM) method was used to impute missing values. To ensure reproducibility, a random seed (seed = 123) was set during the imputation process. Since the value ranges of different variables are very different, and some algorithms used need to perform quantitative normalization of data, Z-score is used for data normalization.

#### 2.4.2 Variables Screening

Univariate COX proportional hazards model (COX) regression analysis was used to conduct preliminary screening of all clinical variables in the training set, and variance inflation factor (VIF) was used to test whether multicollinearity existed among clinical variables after screening. In this study, variables with VIF >5 were deleted. The VIF threshold was set to 5, which is a commonly accepted indicator of moderate multicollinearity. This threshold was selected to strike a balance between retaining enough variables and reducing the impact of multicollinearity. To avoid overfitting the model, we use the least absolute shrinkage and selection operator (LASSO) to filter the variables. LASSO regression compresses the coefficients of some unimportant or redundant variables to zero by applying L1 regularization to the coefficients, thereby reducing model complexity and reducing the risk of overfitting. For LASSO regression, the COX proportional hazards model (family = ‘cox’) was used to identify important predictors. The optimal regularization parameter was selected using the (cv.glmnet) function from the glmnet package in R, with 10-fold cross-validation. The maximum number of iterations (maxit) was set to 1000, and a fixed random seed (seed = 1234) was applied to ensure reproducibility. Random survival forest (RSF) was also used to screen clinical variables, selecting the top 15 variables in order of importance. In this study, after using LASSO and RSF to screen variables, the intersection of the two is taken as the target variable for modeling. The relationship between the selected variables and the outcome was analyzed by restricted cubic spline (RCS).

#### 2.4.3 Model Development

In this study, four ML models were developed to predict the risk of long-term MACEs in PMI patients. They are COX regression, RSF, extreme gradient boosting (XGBoost), and DeepSurv. RSF and XGBoost models are decision tree-based integrated models for classification and regression problems, both of which can efficiently handle high-dimensional datasets with millions of rows and columns. DeepSurv uses deep learning techniques to process survival data, capturing complex non-linear relationships and interaction effects. This makes it more effective than traditional survival analysis methods when dealing with high-dimensional data and complex risk patterns.

According to whether the endpoint appeared or not, 1202 patients were divided into the training set and the testing set according to the ratio of 3:1 by stratified random sampling. The hyperparameters of ML models are optimized by using a grid search method with 5-fold cross-validation. We used the “surv.coxph”, “surv.rfsrc”, “surv.xgboost.cox”, and “surv.deepsurv” learners from the “mlr3extralearners” package to construct the COX proportional hazards model, RSF, XGBoost, and DeepSurv models, respectively. We have included the final selected hyperparameter results in **Supplementary Table 1 **for reference.

#### 2.4.4 Model Performance Evaluation

The concordance index (C-index) or time-dependent AUC was used to evaluate the discrimination of the model, that is, the ability to correctly classify the occurrence of MACEs. Discrimination is an important indicator for evaluating prediction models, especially when screening high-risk populations. The model correction was evaluated using the Brier score. Brier score measures the degree of calibration in a quantitative way and is an indicator used to evaluate the performance of the calibration curve. If the model’s predicted probability is close to the frequency of actual events, the Brier score value will be low, indicating that the model is well calibrated. The predictive benefits of the models were evaluated using the decision curve analysis (DCA). Finally, the best performance model was selected from the four models for the prediction and risk stratification of PMI patients. Using the maximum approximate boarding index calculated by the optimal model as the optimal critical value, PMI patients were divided into high-risk group and low-risk group, and then Log-rank test was used to evaluate whether there were differences in Kaplan-Meier curve between the two groups. To visualize the results of the RSF model, a risk calculator for distant MACEs in PMI patients was developed using the “shiny” package. The SHapley Additive exPlanations (SHAP) value of individual samples is calculated using the “survex” package. The goal of SHAP is to explain the prediction of an instance by calculating the contribution of each feature to the prediction, quantifying the contribution of each feature to the prediction made by the model.

#### 2.4.5 Statistical Analysis

All analyses and calculations were performed using R 4.4.1 and SPSS 26.0 (IBM Corp., Armonk, NY, USA). The continuous data of normal distribution were expressed as mean ± standard deviation (SD), the comparison between the two groups was performed by independent student *t*-test, the continuous data of skewness distribution were expressed by median and quartile [M (Q1, Q3)], and the comparison between the two groups was performed by Mann Whitney U test. The categorical data were expressed as frequency and percentage (n, %), and the comparison between the two groups was made by the Chi-square test or Fisher exact probability method (when the theoretical frequency <1 or the number of cases <40). All *p*-values were two-sided and if below 0.05 the results were considered statistically significant.

## 3. Results

### 3.1 Baseline Characteristics

A total of 1202 patients were enrolled, of whom 1094 (91.0%) were males and 108 (9.0%) were females, and the median age of all patients was 42 (37, 44) years. The median follow-up period was 26 months, ending in June 2023. During the follow-up, a total of 200 patients (16.6%) developed MACEs, including 19 cases of all-cause deaths (9.5%), 8 cases of non-fatal strokes (4.0%), 35 cases of readmissions due to heart failure (17.5%), 75 cases of non-fatal recurrent myocardial infarction (37.5%) and 63 cases of unplanned coronary revascularization (31.5%). Table 1 shows the baseline characteristics and results of 34 clinical variables after univariate COX regression screening. All baseline characteristics of the patients are shown in **Supplementary Table 2**.

**Table 1.** **Baseline characteristics of screening variables and results of univariate COX regression analysis**.

Variables	Total	Non-MACEs	MACEs	*p*	Univariate COX regression	
	(n = 1202)	(n = 1002)	(n = 200)		HR (95% CI)	*p*
BMI (kg/m^2^)	26 (24.4, 27.8)	26 (24.2, 27.7)	27.6 (25.3, 28.7)	<0.001	1.06 (1.03, 1.10)	<0.001
Heart rate (bpm)	75.0 (67.0, 86.0)	75.0 (66.0, 85.0)	77.5 (70.0, 86.0)	0.035	1.01 (1.00, 1.02)	0.024
Diabetes	262 (21.8)	200 (20.0)	62 (31.0)	<0.001	1.62 (1.20, 2.19)	0.002
Killip ≥II	51 (4.2)	34 (3.4)	17 (8.5)	0.002	2.61 (1.59, 4.30)	<0.001
Cardiac shock	11 (0.9)	5 (0.5)	6 (3.0)	0.004	6.09 (2.70, 13.74)	<0.001
IABP	82 (6.8)	60 (6.0)	22 (11.0)	0.016	1.98 (1.27, 3.09)	0.002
Ventilator	17 (1.4)	11 (1.1)	6 (3.0)	0.049	2.42 (1.07, 5.46)	0.033
LAD	947 (78.8)	777 (77.5)	170 (85.0)	0.024	1.60 (1.08, 2.36)	0.018
LM	90 (7.5)	68 (6.8)	22 (11.0)	0.055	1.74 (1.11, 2.71)	0.015
Three diseased vessel	358 (29.8)	281 (28.0)	77 (38.5)	0.004	1.54 (1.16, 2.05)	0.003
PCI therapy	1006 (83.7)	849 (84.7)	157 (78.5)	0.038	0.64 (0.46, 0.90)	0.011
Syntax score	16.0 (11.0, 22.0)	16.0 (11.0, 22.0)	17.5 (12.0, 22.0)	0.021	1.02 (1.00, 1.04)	0.014
WBC (×10^9^/L)	10.2 (8.5, 12.4)	10.1 (8.4, 12.3)	10.6 (8.8, 12.8)	0.037	1.05 (1.01, 1.10)	0.024
ANC (×10^9^/L)	7.5 (5.7, 9.7)	7.4 (5.6, 9.6)	8.0 (5.9, 9.9)	0.059	1.05 (1.01, 1.10)	0.033
ALT (U/L)	42.3 (28.0, 67.8)	42.0 (27.0, 66.0)	43.6 (31.3, 71.6)	0.181	1.11 (1.01, 1.23)	0.038
Urea (mmol/L)	4.3 (3.5, 5.4)	4.3 (3.5, 5.3)	4.7 (3.7, 5.7)	0.008	1.15 (1.07, 1.23) ^*^	<0.001
Cr (µmol/L)	75.0 (66.0, 86.0)	75.0 (66.0, 85.0)	76.0 (65.0, 89.0)	0.358	1.18 (1.07, 1.31) ^*^	0.001
UA (µmol/L)	357.0 (295.0, 429.0)	356.0 (295.0, 422.0)	367.0 (297.0, 462.0)	0.047	1.25 (1.10, 1.43) ^*^	0.001
HbA1c (%)	5.8 (5.6, 6.3)	5.8 (5.6, 6.1)	6.0 (5.8, 7.4)	<0.001	1.16 (1.08, 1.24)	<0.001
Glu (mmol/L)	5.8 (5.1, 7.6)	5.7 (5.0, 7.4)	6.2 (5.1, 9.0)	0.003	1.08 (1.04, 1.12)	<0.001
TyG	9.2 (8.8, 9.7)	9.2 (8.8, 9.7)	9.2 (8.8, 10.0)	0.117	1.24 (1.04, 1.48)	0.018
CRP (mg/L)	5.8 (2.5, 14.5)	5.6 (2.4, 13.7)	6.7 (3.2, 19.4)	0.011	1.16 (1.05, 1.28) ^*^	0.002
TC (mmol/L)	4.8 (4.1, 5.5)	4.8 (4.1, 5.4)	5.0 (4.3, 5.7)	0.008	1.17 (1.05, 1.30)	0.003
LDL-C (mmol/)	3.2 (2.5, 3.8)	3.2 (2.5, 3.8)	3.4 (2.7, 4.0)	0.015	1.18 (1.04, 1.33)	0.008
ApoB (g/L)	1.1 (0.9, 1.3)	1.1 (0.9, 1.3)	1.2 (1.0, 1.4)	0.001	2.17 (1.59, 2.97)	<0.001
FFA (mmol/L)	0.5 (0.5, 0.6)	0.5 (0.5, 0.6)	0.6 (0.5, 0.7)	<0.001	1.84 (1.32, 2.57)	<0.001
D-dimer (mg/L)	0.3 (0.2, 0.4)	0.3 (0.2, 0.4)	0.3 (0.2, 0.5)	0.042	1.16 (1.06, 1.26)	0.002
FIB (mg/dL)	3.3 (2.9, 3.9)	3.3 (2.9, 3.9)	3.5 (3.0, 4.0)	0.009	1.17 (1.05, 1.30)	0.006
LDH (U/L)	434.5 (260.0, 771.3)	418.5 (255.3, 745.3)	490.5 (315.5, 834.8)	0.005	1.24 (1.09, 1.41) ^*^	0.001
CK-MB (U/L)	87.0 (33.0, 181.0)	84.0 (33.0, 174.8)	95.5 (36.8, 197.8)	0.216	1.15 (1.01, 1.31) ^*^	0.029
TNT (ng/mL)	2.0 (0.6, 4.6)	1.9 (0.6, 4.3)	2.6 (1.0, 5.5)	0.007	1.18 (1.04, 1.35) ^*^	0.009
BNP (ng/L)	269.2 (107.6, 689.3)	269.2 (104.7, 630.0)	368.1 (146.7, 1112.0)	<0.001	1.31 (1.21, 1.43) ^*^	<0.001
LVEF (%)	53.0 (46.0, 57.0)	53.0 (47.0, 57.0)	50.0 (43.0, 56.0)	<0.001	0.96 (0.94, 0.97)	<0.001
Diuretics	129 (10.7)	90 (9.0)	39 (19.5)	<0.001	2.33 (1.64, 3.31)	<0.001

Notes: Values are Median (Q1, Q3) or n (%). ^*^HR and 95% CI calculated from standardized data. BMI, body mass index; IABP, intra-aortic balloon pump; LAD, left anterior descending coronary artery; LM, left main coronary artery; PCI, percutaneous coronary intervention; WBC, white blood cell count; ANC, absolute neutrophil count; ALT, alanine aminotransferase; Cr, creatinine; UA, uric acid; HbA1c, glycated hemoglobin; Glu, glucose; TyG, triglyceride-glucose; CRP, c-reactive protein; TC, total cholesterol; LDL-C, low-density lipoprotein cholesterol; ApoB, apolipoprotein B; FFA, free fatty acid; FIB, fibrinogen; LDH, lactate dehydrogenase; CK-MB, creatine kinase MB; TNT, troponin T; BNP, brain natriuretic peptide; LVEF, left ventricular ejection fraction; MACEs, major adverse cardiovascular events; HR, hazard ratio.

### 3.2 Variables Screening

The multicollinearity analysis of 34 meaningful variables in the baseline table showed that the VIF of white blood cell count (WBC), absolute neutrophil count (ANC), total cholesterol (TC), low-density lipoprotein cholesterol (LDL-C) were >5. After removing these variables, the remaining 30 variables were further screened.

**Fig. 2.**
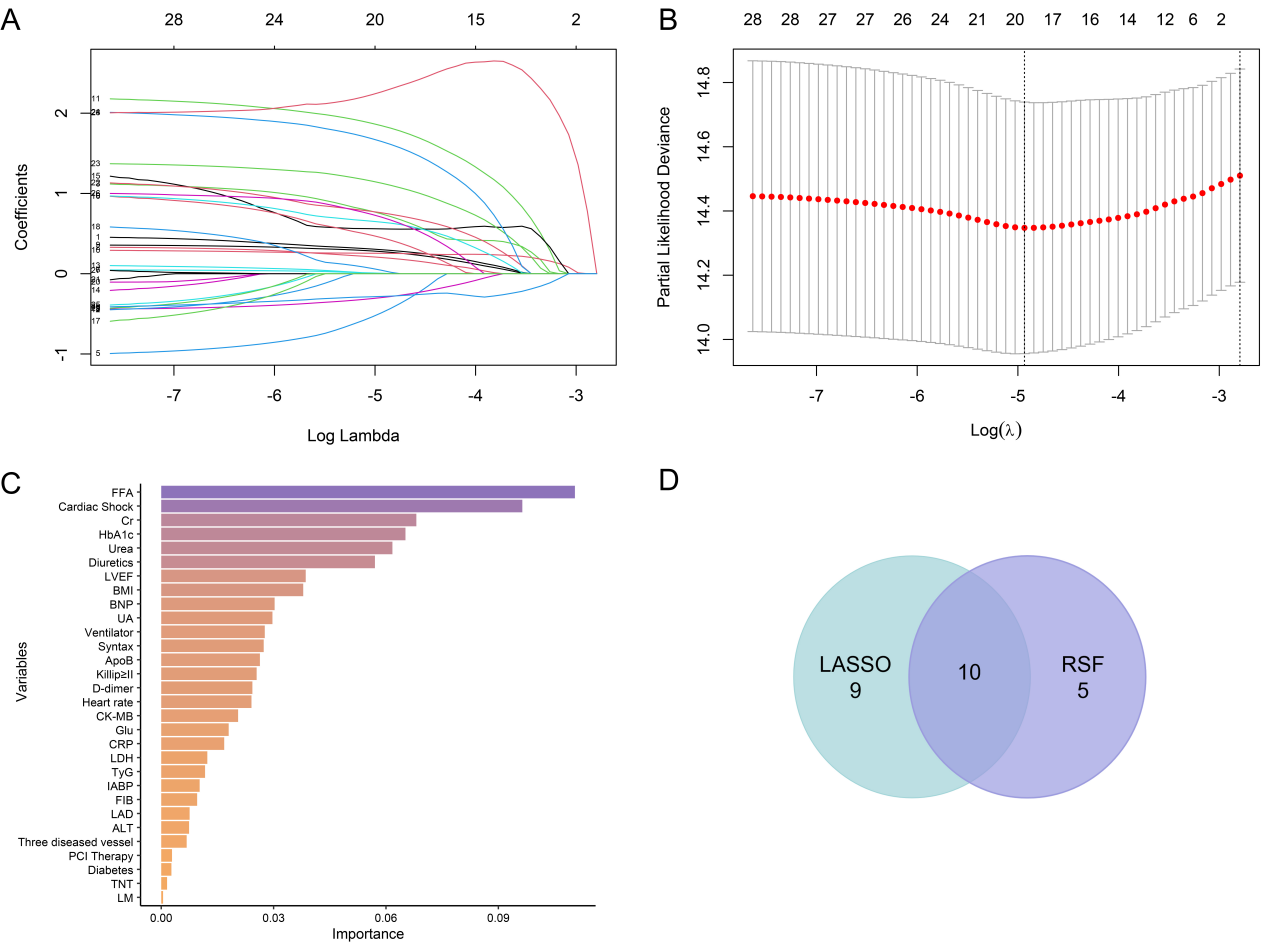
**The process of variable selection**. (A) LASSO coefficient path diagram. The horizontal axis shows the log-transformed regularization parameter (Log(λ)), and the vertical axis represents variable coefficients. As λ increases, stronger penalties shrink more coefficients to zero, highlighting the most relevant variables. (B) LASSO cross-validation plot. The horizontal axis shows Log(λ), and the vertical axis represents the partial likelihood deviance. Red dots represent deviance values from 10-fold cross-validation, with error bars indicating standard error. The left dashed line marks λ_min (minimum deviance), and the right dashed line marks λ_1se (a simpler model within one standard error of λ_min). (C) Ranking the importance of variables in the random survival forest (RSF) model. (D) Venn diagram of the intersection of variables screened by RSF and LASSO. LASSO, least absolute shrinkage and selection operator; ApoB, apolipoprotein B.

The LASSO coefficient path diagram is drawn to show how the coefficients of each variable change under different regularization intensities (Fig. 2A), and the cross-validation diagram (Fig. 2B) shows the performance of the model under different Log Lambda values. Two lambda values were reported for LASSO regression: lambda.min = 0.007193123 and lambda.1se = 0.06112381. After careful consideration, we chose lambda.min because it offers the best predictive performance, even though it retains more variables and results in a slightly more complex model. A total of 19 variables were screened by LASSO, namely diabetes, Killip ≥II, cardiac shock, intra-aortic balloon pump (IABP), left anterior descending coronary artery (LAD), PCI Therapy, three diseased vessels, diuretics, BMI, heart rate, glycated hemoglobin (HbA1c), c-reactive protein (CRP), uric acid (UA), ApoB, free fatty acid (FFA), fibrinogen (FIB), CK-MB, BNP, and left ventricular ejection fraction (LVEF). After RSF selection, the top 15 important variables were selected, which were FFA, cardiac shock, creatinine (Cr), HbA1c, urea, diuretics, LVEF, BMI, BNP, UA, Ventilator, Syntax, ApoB, Killip ≥II, D-dimer (Fig. 2C).

Finally, the first 15 variables ranked by RSF feature importance and the 19 variables selected by LASSO were intersected to obtain 10 variables for modeling (Fig. 2D). The 10 variables were BMI, ApoB, FFA, UA, HbA1c, BNP, LVEF, cardiac shock, Diuretics, and Killip ≥II.

### 3.3 RCS Explores the Relationship between Independent Variables and MACEs

**Fig. 3.**
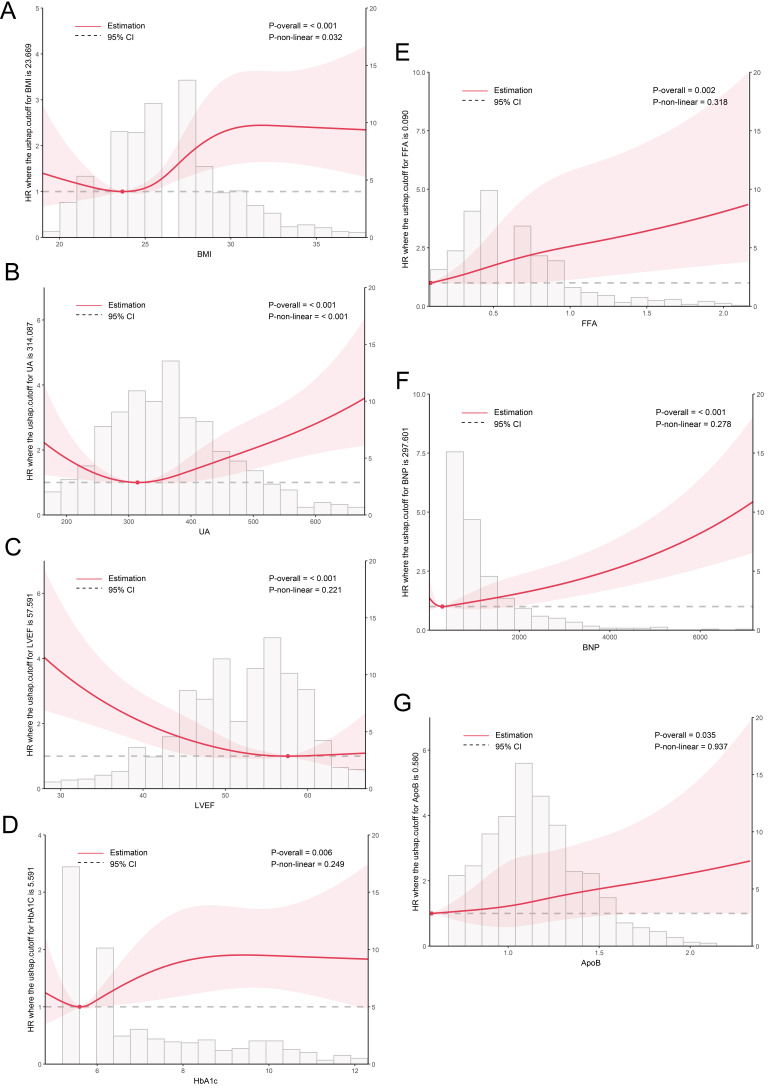
**RCS plot of the final screening variables**. (A) BMI; (B) UA; (C) LVEF; (D) HbA1c; (E) FFA; (F) BNP; (G) ApoB. The relationship between the independent variable and MACEs is shown, as well as the HR and its confidence interval (the red-shaded part in the figure, when the red-shaded part crosses 1, it means that the HR value is meaningless).

The RCS graph graphically shows how the independent variable affects the hazard ratio value (HR value) and thus the occurrence of MACEs in different value intervals. In this study, RCS analysis was carried out on continuous variables among the 10 selected variables (Fig. 3), and the results showed that BMI, UA and MACEs showed a roughly J-shaped relationship: When BMI >23.669 kg/m^2^, MACEs risk increased with the increase of BMI value, and the lowest BMI estimate of MACEs risk was 23.669 kg/m^2^. When UA >314.087 µmol/L, MACEs risk increased with the increase of UA value and the lowest UA estimate of MACEs risk was estimated to be 314.087 µmol/L. The relationship between the remaining variables ApoB, FFA, HbA1c, BNP, LVEF and MACEs were roughly linear.

### 3.4 Model Development and Performance Evaluation

**Fig. 4.**
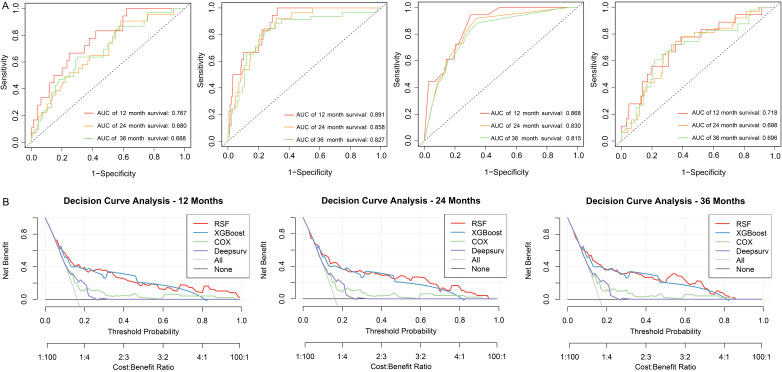
**Performance evaluation of four models**. (A) Receiver operating characteristic (ROC) curves of different models at 12, 24, and 36 months. From left to right are COX regression, Random Survival Forest, XGBoost, and DeepSurv models. (B) Decision curve analysis of four models at 12 months, 24 months and 36 months.

The performance of the four models was comprehensively evaluated using several metrics, including discrimination (AUC and C-index), calibration (Brier Score), and clinical utility (DCA). First, the discrimination of the four models was evaluated by AUC and C-index (Fig. 4A). The RSF model consistently outperforming others across all time points (12-month: 0.891; 24-month: 0.858; 36-month: 0.827). These high AUC values highlight the RSF model’s excellent ability to identify high-risk individuals. Additionally, its C-index of 0.815 further confirms strong predictive reliability, with values above 0.8 considered very good for risk stratification. Second, the RSF model achieved an average Brier score of 0.125, which was superior to the other models (Table 2). A lower Brier score indicates better overall performance, as it reflects both the accuracy of the predicted probabilities and their alignment with actual outcomes. Last, DCA demonstrated that the RSF model provided the highest net benefit across a range of threshold probabilities at 12 months, 24 months, and 36 months, outperforming the XGBoost, COX regression, and DeepSurv models (Fig. 4B). Particularly in the lower threshold probability range, where identifying high-risk individuals is essential for early intervention, the RSF model exhibited significant advantages. This underscores its clinical utility and potential to guide personalized treatment strategies. The baseline characteristics of the training and testing sets used for the RSF model are shown in **Supplementary Table 3**. Statistical analysis revealed no significant differences in variable distributions between the two datasets, ensuring balanced training and testing set. In conclusion, the RSF model was chosen as the primary tool for risk prediction in this study due to its superior discrimination, calibration, and clinical utility.

**Table 2.** **Evaluation index of the ML models**.

Indicators	COX	RSF	XGBoost	DeepSurv
12-month AUC	0.767	0.891	0.868	0.718
24-month AUC	0.680	0.858	0.830	0.698
36-month AUC	0.788	0.827	0.815	0.696
C-index	0.685	0.815	0.803	0.683
Brier	0.149	0.125	0.156	0.388

Notes: AUC, area under the curve; ML, machine learning.

### 3.5 Risk Stratification Based on the RSF Model

**Fig. 5.**
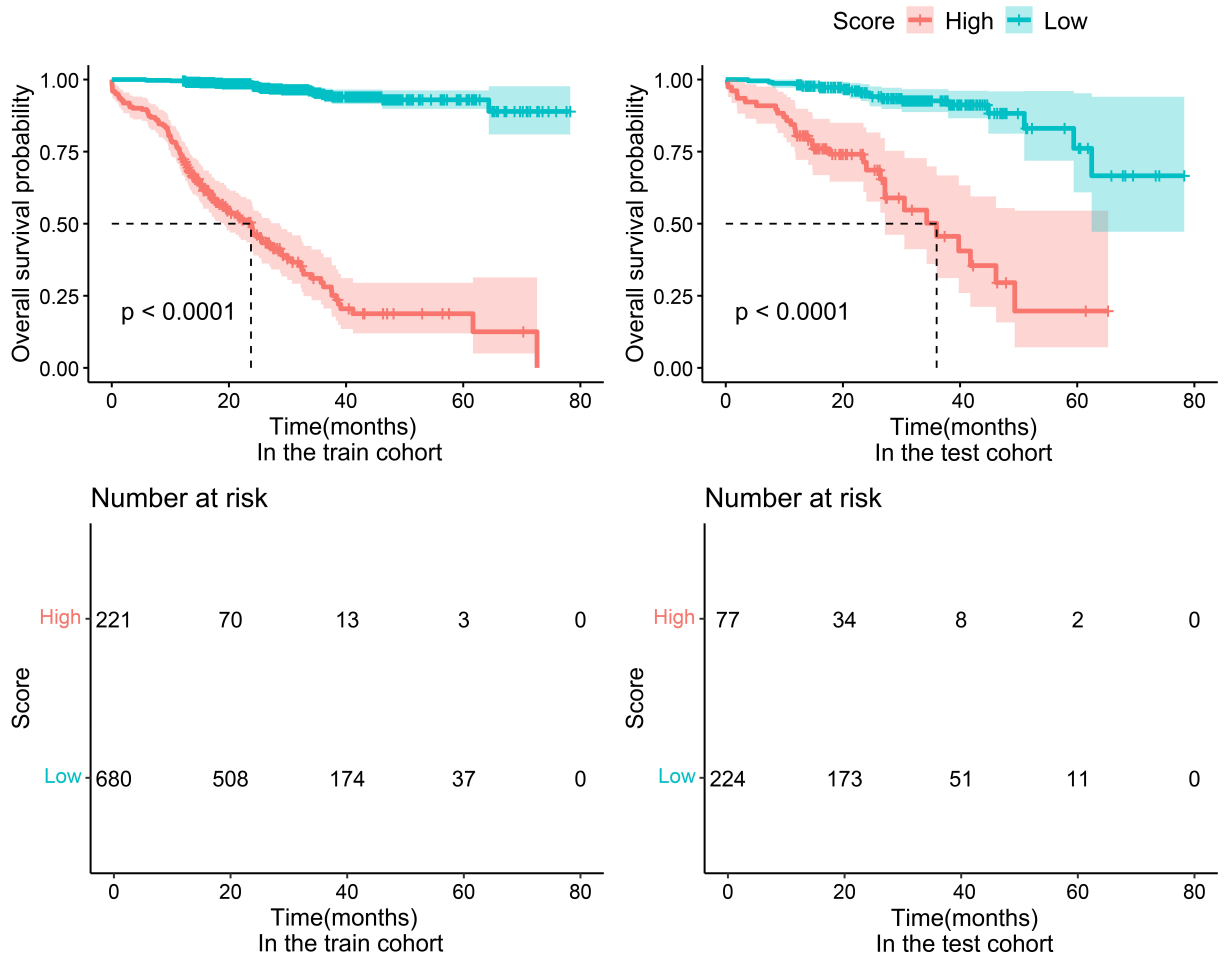
**Survival analysis of high-risk and low-risk populations in the RSF model**. Kaplan-Meier curves for the training set (left) and testing sets (right), and the number of people who did not develop MACEs over time.

The RSF model was used to predict and stratify the risk of MACEs in PMI patients. Taking the risk score (24.90 scores) corresponding to the maximal Youden’s index as the optimal cut-off value, patients were divided into a high-risk group and a low-risk group, as shown in the Kaplan-Meier curve (Fig. 5), in both the training set and the testing set, the incidence of MACEs was more pronounced in high-risk patients (the Log-rank test showed a significant difference between the two groups, *p* < 0.0001), and special attention needs to be paid to the management and intervention of patients in the high-risk group in clinical practice.

### 3.6 Importance Ranking of Variables and Forest Map

**Fig. 6.**
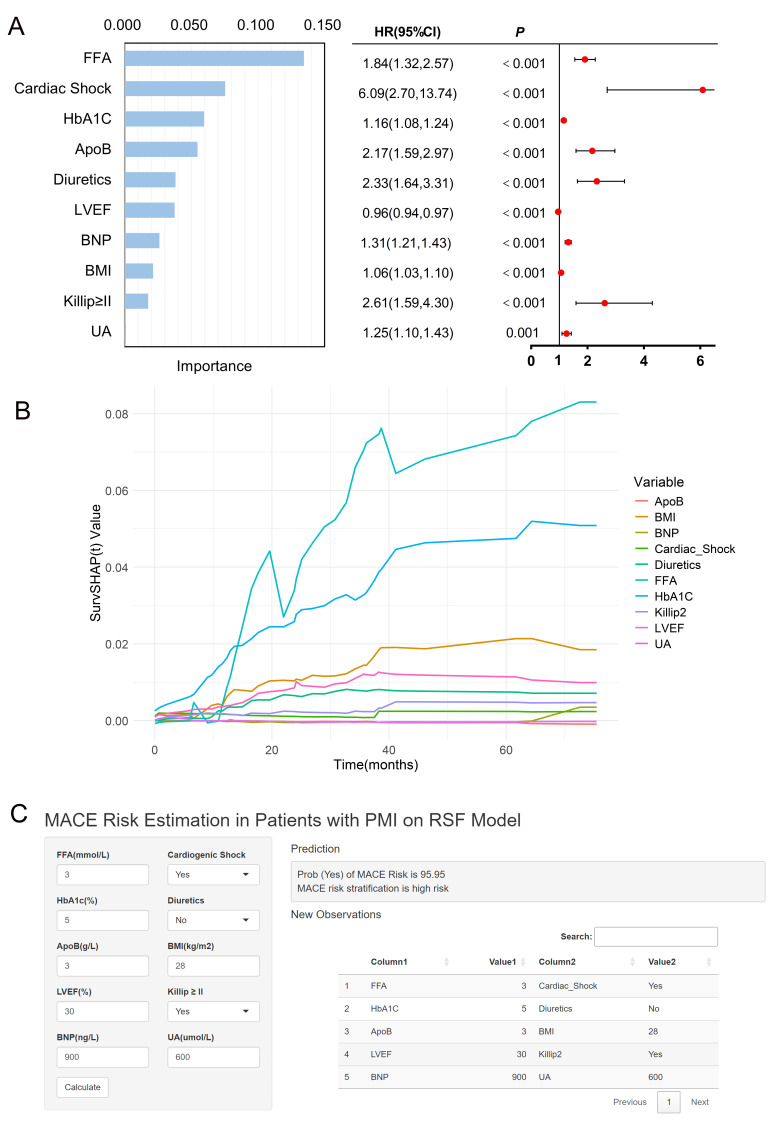
**Feature importance ranking, SurvSHAP(t) prediction interpretation analysis and risk calculator**. (A) Feature importance ranking of RSF model and forest plot. (B) The figure shows the temporal changes in the SurvSHAP(t) values of the key variables in the RSF model. The x-axis represents time (in months), while the y-axis represents the magnitude of the SurvSHAP(t) value, which quantifies the contribution of each variable to the prediction of survival at a given time point. The figure shows how the predicted survival probability of a single sample at different time points is affected by various features. (C) Risk calculator for MACEs in patients with PMI. The left page inputs the value of the variable, and the right page outputs the risk score and level.

Fig. 6A shows the 10 most important clinical variables in the RSF model, ranked in order of importance, namely FFA, cardiogenic shock, HbA1c, ApoB, diuretics, LVEF, BNP, BMI, Killip ≥II, and UA. The bar chart on the left shows the relative importance of each variable. The forest plot on the right shows the association between each variable and the risk of MACEs. In Fig. 6B, the temporal contributions of individual variables to survival predictions are depicted using SurvSHAP(t) values. Among all variables, FFA exhibits the highest influence on survival predictions, with a consistent upward trend over time, reaching its peak contribution at approximately 60 months. In contrast, BNP and BMI show moderate but stable contributions throughout the timeline. Variables such as Cardiac_Shock, Diuretics, and HbA1c demonstrate smaller contributions with relatively flat or minimal temporal variations. The results highlight the dominant role of FFA in the survival prediction of the RSF model for this individual.

### 3.7 Model Visualization

To facilitate the use of prognostic models in clinical management, we developed a risk calculator based on the Shiny program package. The left side of the page (Fig. 6C) allows the user to enter each clinical characteristic, and the right side of the page calculates the predicted probability of distant MACEs and risk stratification based on information about PMI patients.

## 4. Discussion

This study developed and validated an interpretable ML risk prediction model for predicting the risk of long-term MACEs in PMI patients and analyzed clinical variables that influence the development of MACEs. The evaluation results of comprehensive discrimination, calibration and clinical utility showed that the RSF model performed best. Using the risk score (24.90) calculated by the RSF model as the critical value, patients were divided into high-risk group and low-risk group, and there was significant difference in Kaplan-Meier survival analysis curve between the two groups (*p* < 0.0001). The ten clinical variables of feature importance ranking are FFA, cardiac shock, HbA1c, ApoB, Diuretics, LVEF, BNP, BMI, Killip ≥II, and UA. By calculating the risk of MACEs through a risk calculator and explaining individual risk sources and possible intervention directions through SHAP values, it is hoped that personalized and transparent clinical management can be achieved.

The results of this study highlight the superior performance of the RSF model in predicting the risk of MACEs in AMI patients, as evidenced by its discrimination, reliable calibration, and robust clinical utility. Compared with traditional models such as the COX proportional hazards model, RSF overcomes key limitations by capturing complex nonlinear relationships and high-order interactions without relying on the proportional hazards assumption. This adaptability is particularly valuable in real-world clinical scenarios, where these assumptions are often violated [20, 21]. Although XGBoost is a powerful machine learning algorithm, its application to survival data often requires additional modifications, such as implementing COX loss functions, which may introduce constraints, and it also demands extensive parameter tuning [22]. Similarly, DeepSurv, as a deep learning method, requires large-scale datasets to perform optimally and is prone to overfitting with limited data [23]. In contrast, RSF natively supports survival analysis, offering seamless integration, robust performance even with moderate sample sizes, and higher predictive accuracy without the need for extensive modifications or tuning, making it more practical for real-world clinical applications.

Insulin resistance (IR), elevated HbA1c, and metabolic abnormalities such as abnormal BMI, dyslipidemia, and hyperuricemia are critical contributors to myocardial injury and increased MACEs risk, particularly in young PMI patients. IR leads to myocardial damage through impaired diastole, altered glucose utilization, and microvascular dysfunction, while metabolic abnormalities like elevated BMI and dysregulated lipid metabolism trigger inflammation and thrombosis through the release and accumulation of fat metabolites [24, 25]. The J-shaped relationship between BMI and MACEs observed in this study using RCS is consistent with the findings of some other studies [26, 27], Flegal *et al*.’s [28] study has shown that overweight individuals have a lower risk than normal-weight individuals. This study’s findings highlight the strong association of FFA with adverse cardiovascular events, FFA assessment alongside traditional risk factors to identify high-risk individuals requiring closer monitoring and intervention. Dyslipidemia, particularly elevated ApoB-containing lipoproteins such as LDL-C, lipoprotein(a), and triglyceride-rich lipoproteins, significantly contributes to MACEs risk. While LDL-C remains a primary target for lipid management, this study’s RCS analysis aligns with prior research showing a linear relationship between higher ApoB levels and increased MACEs risk, even in patients on high-intensity statin therapy [29, 30, 31]. These findings suggest that for younger PMI patients, early and aggressive management of ApoB levels may be crucial in reducing cardiovascular risk. Elevated uric acid levels also emerged as an important predictor of MACEs. Through mechanisms such as oxidative stress, endothelial dysfunction, and inflammation, uric acid exacerbates insulin resistance and promotes atherosclerosis [32, 33, 34, 35]. Managing uric acid levels may disrupt this pathological cycle, offering an additional avenue for intervention in younger patients.

Cardiac function plays a pivotal role in determining MACEs risk. Variables in the RSF model, such as elevated BNP, reduced LVEF, Killip ≥II, cardiogenic shock, and in-hospital diuretic use, reflect poor cardiac function during hospitalization. This study’s RCS analysis showed a linear relationship between decreasing LVEF and increasing MACEs risk, consistent with previous research linking reduced ejection fraction with poorer outcomes in PCI-treated patients [36, 37]. These findings emphasize the importance of targeted cardiac rehabilitation and monitoring strategies for PMI patients with compromised cardiac function.

A comprehensive strategy is essential for younger PMI patients to reduce MACEs risk, combining advanced predictive tools and tailored management interventions. Aggressive control of ApoB, FFAs, and uric acid levels is crucial to address inflammation, thrombosis, and oxidative stress, while individualized BMI management mitigates the J-shaped risk relationship observed with MACEs. Targeted cardiac rehabilitation and monitoring of BNP and LVEF further enhance outcomes in patients with compromised cardiac function. The RSF model demonstrated its strength by integrating these multifactorial risks into a comprehensive predictive framework. With the addition of SHAP values, the model provides individual-level explanations, helping clinicians identify key contributing factors for each patient’s risk. Combined with a personalized risk calculator, these tools enable dynamic and patient-specific intervention strategies targeting modifiable risk factors such as IR, dyslipidemia, hyperuricemia, and cardiac dysfunction. This approach supports more effective prevention and treatment, ultimately improving long-term outcomes and reducing MACEs incidence in young PMI patients.

This study has several limitations. Most importantly, it lacks external validation with independent cohorts, which is essential for confirming the generalizability and robustness of the algorithm. In future research, we will incorporate patients from diverse regions and hospitals to perform external validation, ensuring broader applicability across different populations. Additionally, this study primarily focuses on clinical characteristics, missing key factors such as lifestyle, dietary habits, and multi-omics markers. Expanding these variables in future studies could provide a more comprehensive understanding of risk factors and enhance the predictive accuracy of the model.

## 5. Conclusions

The RSF-based risk stratification tool demonstrated excellent performance, proving its capability to accurately predict MACEs risk in PMI patients. The model identified critical predictors such as FFA, cardiogenic shock, HbA1c, ApoB, diuretic use, LVEF, BNP, BMI, Killip ≥II, and UA, highlighting the multifactorial complexity of MACEs risk. Enhanced by SHAP values and a risk calculator, the RSF model provides a personalized framework to identify high-risk patients, pinpoint key risk factors, and guide targeted interventions. This approach enables early management of modifiable risks, improving outcomes and reducing MACEs in PMI patients.

## Data Availability

The datasets used and/or analysed during the current study are available from the corresponding author on reasonable request.

## Abbreviations

ACS, acute coronary syndrome; ALT, alanine aminotransferase; ANC, absolute neutrophil count; ApoB, apolipoprotein B; AUC, area under the curve; BNP, brain natriuretic peptide; BMI, body mass index; C-index, concordance index; CABG, coronary artery bypass grafting; CK-MB, creatine kinase MB; COX, COX proportional hazards model; Cr, creatinine; CRP, c-reactive protein; DCA, decision curve analysis; ECG, Electrocardiogram; FFA, free fatty acid; FIB, fibrinogen; Glu, glucose; HbA1c, glycated hemoglobin; HR value, hazard ratio value; IABP, intra-aortic balloon pump; LAD, left anterior descending coronary artery; LASSO, least absolute shrinkage and selection operator; LM, left main coronary artery; LDH, lactate dehydrogenase; LDL-C, low-density lipoprotein cholesterol; LVEF, left ventricular ejection fraction; MACEs, major adverse cardiovascular events; ML, machine learning; PCI, percutaneous coronary intervention; PMI, premature myocardial infarction; RCS, restricted cubic spline; ROC, receiver operating characteristic; RSF, random survival forest; SD, standard deviation; SHAP, SHapley Additive exPlanations; ST-T, ST segment and T wave; TC, total cholesterol; TNT, troponin T; TyG, triglyceride-glucose; UA, uric acid; VIF, variance inflation factor; WBC, white blood cell count; XGBoost, extreme gradient boosting.

## Supplementary Material

Supplementary material associated with this article can be found, in the online version, at https://doi.org/10.31083/RCM31298.
