# Supplementary material for: Using Machine Learning to Predict MACEs Risk in Patients with Premature Myocardial Infarction
Source: Rev Cardiovasc Med. 2025 May 20;26(5):31298. doi: 10.31083/RCM31298 (PMC12135653; doi:10.31083/RCM31298)
Supplement: Supplementary file 1 [file 2153-8174-26-5-31298-s1.docx]

**Supplementary Table 1**. Model hyperparameters

| **Model** | hyperparameter |  |
| --- | --- | --- |
| **RSF** | ntree | 450 |
|  | mtry | 14 |
|  | nodesize | 14 |
|  | nodedepth | 16 |
| **XGBoost** | gamma | 1 |
|  | alpha | 0.5 |
|  | Lambda  eta | 1  0.0001 |
| **DeepSurv** | num_layers | 11 |
|  | num_nodes_per_layer | 49 |
|  | betas | 0.2 |
|  | alpha | 0.1 |
|  | batch_size | 33 |

RSF, random survival forests; XGBoost, extreme gradient boosting.

**Supplementary Table 2.** Baseline characteristics of PMI patients

| Variables | Total  (n = 1202) | Non-MACE  (n = 1002) | MACE  (n = 200) | *P* |
| --- | --- | --- | --- | --- |
| Male | 1094 (91.0) | 920 (91.8) | 174 (87.0) | 0.041 |
| Age (year) | 42 (37, 44) | 42 (37, 44) | 43 (37, 45) | 0.148 |
| BMI (kg/m^2^) | 26.0 (24.4, 27.8) | 26.0 (24.2, 27.7) | 27.6 (25.3, 28.7) | < 0.001 |
| **Personal history and past medical history** | | | | |
| Smoke | 844 (70.2) | 706 (70.5) | 138 (69.0) | 0.743 |
| Alcohol | 447 (37.2) | 372 (37.1) | 75 (37.5) | 0.984 |
| Diabetes | 262 (21.8) | 200 (20.0) | 62 (31.0) | < 0.001 |
| Hypertension | 578 (48.1) | 474 (47.3) | 104 (52.0) | 0.256 |
| Hyperlipidemia | 299 (24.9) | 260 (26.0) | 39 (19.5) | 0.066 |
| CKD | 16 (1.3) | 12 (1.2) | 4 (2.0) | 0.322 |
| Stroke | 43 (3.6) | 33 (3.3) | 10 (5.0) | 0.328 |
| Family history of CAD | 132 (11.0) | 110 (11.0) | 22 (11.0) | 1.000 |
| **Admission signs** | | | | |
| HR (bpm) | 75 (67, 86) | 75 (66, 85) | 77.5 (70, 86) | 0.035 |
| SBP (mmHg) | 130 (120, 143) | 130 (120, 142) | 130 (120, 145) | 0.567 |
| DBP (mmHg) | 80 (70, 90) | 80 (70, 90) | 80 (72, 90) | 0.992 |
| SI | 0.6 (0.5, 0.7) | 0.6 (0.5, 0.7) | 0.6 (0.5, 0.7) | 0.144 |
| STEMI | 929 (77.3) | 776 (77.5) | 153 (76.5) | 0.842 |
| Killip≥II | 51 (4.2) | 34 (3.4) | 17 (8.5) | 0.002 |
| Cardiogenic shock | 11 (0.9) | 5 (0.5) | 6 (3.0) | 0.004 |
| IABP | 82 (6.8) | 60 (6.0) | 22 (11.0) | 0.016 |
| Ventilator | 17 (1.4) | 11 (1.1) | 6 (3.0) | 0.049 |
| Cardiac Arrest | 24 (2.0) | 19 (1.9) | 5 (2.5) | 0.579 |
| **Diseased vessel** | | | | |
| LAD | 947 (78.8) | 777 (77.5) | 170 (85.0) | 0.024 |
| LCX | 616 (51.3) | 502 (50.1) | 114 (57.0) | 0.088 |
| RCA | 678 (56.4) | 557 (55.6) | 121 (60.5) | 0.230 |
| LM | 90 (7.5) | 68 (6.8) | 22 (11.0) | 0.055 |
| Three diseased vessels | 358 (29.8) | 281 (28.0) | 77 (38.5) | 0.004 |
| Coronary thrombosis | 336 (28.0) | 281 (28.0) | 55 (27.5) | 0.944 |
| Occluded vessel | 725 (60.3) | 604 (60.3) | 121 (60.5) | 1.000 |
| Syntax score | 16 (11, 22) | 16 (11, 22) | 18 (12, 22) | 0.021 |
| PCI Therapy | 1006 (83.7) | 849 (84.7) | 157 (78.5) | 0.038 |
| **Laboratory test results** | | | | |
| WBC (×10^9^/L) | 10.2 (8.5, 12.4) | 10.1 (8.4, 12.3) | 10.6 (8.8, 12.8) | 0.037 |
| ANC (×10^9^/L) | 7.5 (5.7, 9.7) | 7.35 (5.6, 9.6) | 8.0 (5.9, 9.9) | 0.059 |
| ALT (U/L) | 42.3 (28.0, 67.8) | 42.0 (27.0, 66.0) | 43.6 (31.3, 71.6) | 0.181 |
| AST (U/L) | 104.8 (44.2, 214.3) | 103.1 (43.4, 212.6) | 122.85 (51.4, 238.2) | 0.122 |
| γ-GT (U/L) | 37.0 (25.0, 57.0) | 36.1 (24.8, 56.0) | 41.4 (25.5, 61.7) | 0.114 |
| Urea (mmol/L) | 4.3 (3.5, 5.4) | 4.3 (3.5, 5.3) | 4.7 (3.7, 5.7) | 0.008 |
| Cr (umol/L) | 75.0 (66.0, 86.0) | 75 (66.0, 85.0) | 76 (65.0, 89.0) | 0.358 |
| UA (umol/L) | 357 (295, 429) | 356 (295, 422) | 367 (297, 462) | 0.047 |
| HbA1C (%) | 5.8 (5.6, 6.3) | 5.8 (5.6, 6.1) | 6.0 (5.8, 7.4) | < 0.001 |
| Glu (mmol/L) | 5.8 (5.1, 7.6) | 5.7 (5.0, 7.4) | 6.2 (5.1, 9.0) | 0.003 |
| TyG | 9.2 (8.8, 9.7) | 9.2 (8.8, 9.7) | 9.2 (8.8, 10.0) | 0.117 |
| CRP (mg/L) | 5.8 (2.5, 14.5) | 5.6 (2.4, 13.7) | 6.7 (3.2, 19.4) | 0.011 |
| HCY (μmol/L) | 12.8 (10.2, 18.8) | 12.8 (10.4, 19.0) | 12.8 (9.6, 17.2) | 0.205 |
| TBA (μmol/L) | 1.4 (0.9, 2.4) | 1.4 (0.9, 2.4) | 1.5 (0.9, 2.4) | 0.943 |
| TC (mmol/L) | 4.8 (4.1, 5.5) | 4.8 (4.1, 5.4) | 5.0 (4.3, 5.7) | 0.008 |
| TG (mmol/L) | 2.0 (1.4, 2.9) | 2.0 (1.4, 2.9) | 2.0 (1.4, 3.0) | 0.881 |
| LDL-C (mmol/L) | 3.2 (2.5, 3.8) | 3.2 (2.5, 3.8) | 3.4 (2.7, 4.0) | 0.015 |
| HDL-C (mmol/L) | 0.9 (0.8, 1.1) | 0.9 (0.8, 1.1) | 0.9 (0.8, 1.1) | 0.665 |
| VLDC-L (mmol/L) | 0.6 (0.4, 0.8) | 0.6 (0.4, 0.8) | 0.5 (0.4, 0.8) | 0.645 |
| FC (mmol/L) | 0.6 (0.4, 0.8) | 0.6 (0.4, 0.8) | 0.5 (0.4, 0.8) | 0.724 |
| ApoA1 (g/L) | 1.1 (1.0, 1.2) | 1.1 (1.0, 1.2) | 1.1 (1.0, 1.3) | 0.153 |
| ApoB (g/L) | 1.1 (0.9, 1.3) | 1.1 (0.9, 1.3) | 1.2 (1.0, 1.4) | 0.001 |
| Lpa (mg/dl) | 43.4 (14.9, 116.9) | 43.4 (15.1, 121.3) | 42.1 (14.5, 104.7) | 0.371 |
| FFA (mmol/L) | 0.5 (0.5, 0.6) | 0.5 (0.5, 0.6) | 0.6 (0.5, 0.7) | < 0.001 |
| D-dimer (mg/L) | 0.3 (0.2, 0.4) | 0.3 (0.2, 0.4) | 0.3 (0.2, 0.5) | 0.042 |
| FIB (mg/dL) | 3.3 (2.9, 3.9) | 3.3 (2.9, 3.9) | 3.5 (3.0, 4.0) | 0.009 |
| CK (U/L) | 1006 (324, 2086) | 968 (323, 2086) | 1198 (366, 2123) | 0.307 |
| CK-MB (U/L) | 87 (33, 181) | 84 (33, 175) | 96 (37, 198) | 0.216 |
| LDH (U/L) | 435 (260, 771) | 419 (255, 745) | 490 (316, 835) | 0.005 |
| TNT (ng/mL) | 2.0 (0.6, 4.5) | 1.9 (0.6, 4.3) | 2.6 (1.0, 5.5) | 0.007 |
| BNP (ng/L) | 269 (108, 689) | 269 (105, 630) | 368 (147, 1112) | < 0.001 |
| **Echocardiography** | | | | |
| LA (mm) | 36 (34, 39) | 36 (34, 39) | 37 (35, 39) | 0.343 |
| LV (mm) | 52 (49, 55) | 52 (49, 55) | 52 (49, 56) | 0.083 |
| LVEF (%) | 53 (46, 57) | 53 (47, 57) | 50 (43, 56) | < 0.001 |
| **Admission medication** | | | | |
| Aspirin | 1201(99.9) | 1001(99.9) | 200(100.0) | 0.655 |
| P2Y12 inhibitors | 1200(99.8) | 1000(99.8) | 200(100.0) | 0.527 |
| Statin | 1182 (98.3) | 987 (98.5) | 195 (97.5) | 0.358 |
| ACEI/ARB | 830 (69.1) | 685 (68.4) | 145 (72.5) | 0.284 |
| Beta Blockers | 976 (81.2) | 814 (81.2) | 162 (81.0) | 1.000 |
| Diuretics | 129 (10.7) | 90 (9.0) | 39 (19.5) | < 0.001 |

Values are median (Q1, Q3) or n (%).

CKD, chronic kidney disease; CAD, coronary heart disease; AMI, acute myocardial infarction; IABP, intra-aortic balloon refutation; LAD, **left anterior descending** coronary artery; LCX, left circumflex coronary artery; RCA, right coronary artery; LM, left main coronary artery; PCI, percutaneous coronary intervention; BMI, body mass index; HR, heart rate; SBP, systolic blood pressure; DBP, diastolic blood pressure; SI, shock index; WBC, white blood cell count; ANC, absolute neutrophil count; ALT, **alanine aminotransferase**; AST, aspartate aminotransferase; γ-GT, **γ-glutamyl transpeptidase;** Cr, creatinine; UA, uric acid; HbA1c, glycated hemoglobin; Glu, glucose; TyG, **triglyceride-glucose index;** CRP, **C-reactive protein;** HCY, **homocysteine;** TBA, total bile acids; **TC, total** cholesterol; TG, triglyceride; LDL-C, low-density lipoprotein cholesterol; HDL-C, high-density lipoprotein cholesterol; VLDL-C, very low-density lipoprotein cholesterol; FC, free cholesterol; ApoA1,apolipoprotein A1; ApoB, apolipoprotein B; Lpa, lipoprotein a; FFA, free fatty acid; FIB, fibrinogen; CK, c**reatine kinase;** CK-MB, **creatine kinase lsoenzyme MB**; LDH, lactate dehydrogenase; TNT, troponin T; BNP, brain natriuretic peptide; LA, **left atrium**; LV, **left ventricle;** LVEF, left ventricular ejection fraction; ACEI/ARB, angiotensin-converting enzyme inhibitors/ angiotensin II receptor antagonist.

**Supplementary Table 3.** Baseline characteristics of the training set and the testing set

| Variables | Training Set (n＝901) | Testing Set (n＝301) | *P* |
| --- | --- | --- | --- |
| MACE | 151 (16.8) | 49 (16.3) | 0.917 |
| Month | 25.83 (16.47, 37.23) | 27.17 (16.97, 37.87) | 0.496 |
| BMI (kg/m^2^) | 26 (24.22, 27.78) | 26 (24.4, 27.78) | 0.609 |
| HR (bpm) | 75 (67, 86) | 75 (66, 86) | 0.576 |
| Diabetes | 202 (22.42) | 60 (19.93) | 0.410 |
| Killip≥2 | 37 (4.1) | 14 (4.7) | 0.810 |
| Cardiac shock | 10 (1.1) | 1 (0.3) | 0.309 |
| IABP | 69 (7.66) | 13 (4.32) | 0.063 |
| Ventilator | 16 (1.8) | 1 (0.3) | 0.088 |
| LAD | 717 (79.6) | 230 (76.4) | 0.279 |
| LM | 65 (7.2) | 25 (8.3) | 0.62 |
| Three diseased vessel | 268 (29.7) | 90 (29.9) | 1.000 |
| PCI Therapy | 764 (84.8) | 242 (80.4) | 0.090 |
| Syntax score | 16 (11, 22) | 16 (11, 20.5) | 0.096 |
| WBC (×10^9^/L) | 10.23 (8.61, 12.46) | 10.12 (8.05, 12.28) | 0.151 |
| ANC (×109/L) | 7.58 (5.8, 9.77) | 7.2 (5.2, 9.4) | 0.070 |
| Urea (mmol/L) | 4.3 (3.5, 5.4) | 4.3 (3.6, 5.4) | 0.791 |
| Cr (μmol/L) | 76 (66, 86) | 75 (66, 86) | 0.648 |
| UA (umol/L) | 356 (292, 428) | 363 (304, 430) | 0.445 |
| HbA1C (%) | 5.8 (5.6, 6.3) | 5.8 (5.5, 6.2) | 0.418 |
| Glu (mmol/L) | 5.76 (5.06, 7.62) | 5.69 (5.01, 7.48) | 0.590 |
| TyG | 9.18 (8.77, 9.7) | 9.14 (8.77, 9.73) | 0.756 |
| CRP (mg/L) | 6.1 (2.63, 14.81) | 5.36 (2.14, 13.61) | 0.278 |
| TC (mmol/L) | 4.78 (4.11, 5.46) | 4.83 (4.13, 5.54) | 0.580 |
| LDLC (mmol/L) | 3.19 (2.54, 3.78) | 3.16 (2.51, 3.83) | 0.829 |
| ApoB(g/L) | 1.12 (0.93, 1.3) | 1.12 (0.95, 1.32) | 0.775 |
| FFA (mmol/L) | 0.54 (0.5, 0.62) | 0.54 (0.54, 0.62) | 0.203 |
| D-dimer (mg/L) | 0.29 (0.22, 0.43) | 0.3 (0.23, 0.43) | 0.164 |
| FIB (mg/dL) | 3.3 (2.89, 3.87) | 3.34 (2.83, 3.96) | 0.700 |
| LDH (U/L) | 454 (261, 790) | 391 (252, 686) | 0.094 |
| CK-MB (U/L) | 90 (34, 181) | 78 (28, 183) | 0.310 |
| TNT (ng/mL) | 1.95 (0.63, 4.6) | 2.04 (0.57, 4.4) | 0.958 |
| BNP (ng/L) | 269.2 (107.88, 689.4) | 284.2 (106.3, 670.7) | 0.899 |
| LVEF (%) | 52.5 (47, 57) | 53 (46, 57) | 0.644 |
| Diuretics | 98 (10.9) | 31 (10.3) | 0.863 |

Values are median (Q1, Q3) or n (%).

MACE: major adverse cardiovascular events; BMI, body mass index; HR, heart rate; IABP, intra-aortic balloon refutation; LAD, **left anterior descending** coronary artery; LM, left main coronary artery; PCI, percutaneous coronary intervention; WBC, white blood cell count; ANC, absolute neutrophil count; ALT, alanine aminotransferase; Cr, creatinine; UA, uric acid; HbA1c, glycated hemoglobin; Glu, glucose; TyG, triglyceride-glucose; CRP, c-reactive protein; TC, total cholesterol; LDL-C, low-density lipoprotein cholesterol; ApoB, apolipoprotein B; FFA, free fatty acid; FIB, fibrinogen; LDH, lactate dehydrogenase; CK-MB, creatine Kinase MB; TNT, troponin T; BNP, brain natriuretic peptide; LVEF, left ventricular ejection fraction.
